# HGF Expressing Stem Cells in Usual Interstitial Pneumonia Originate from the Bone Marrow and Are Antifibrotic

**DOI:** 10.1371/journal.pone.0065453

**Published:** 2013-06-19

**Authors:** Amiq Gazdhar, Njomeza Susuri, Katrin Hostettler, Mathias Gugger, Lars Knudsen, Michael Roth, Matthias Ochs, Thomas Geiser

**Affiliations:** 1 Department of Pulmonary Medicine, University Hospital Bern, Bern, Switzerland; 2 Department of Clinical Research, University of Bern, Bern, Switzerland; 3 Department of Pathology, University of Bern, Bern, Switzerland; 4 Pneumology, Department of Biomedicine University Hospital Basel, Basel, Switzerland; 5 Institute of Functional and Applied Anatomy, Hannover Medical School, Hannover, Germany; University of Pittsburgh, United States of America

## Abstract

**Background:**

Pulmonary fibrosis may result from abnormal alveolar wound repair after injury. Hepatocyte growth factor (HGF) improves alveolar epithelial wound repair in the lung. Stem cells were shown to play a major role in lung injury, repair and fibrosis. We studied the presence, origin and antifibrotic properties of HGF-expressing stem cells in usual interstitial pneumonia.

**Methods:**

Immunohistochemistry was performed in lung tissue sections and primary alveolar epithelial cells obtained from patients with usual interstitial pneumonia (UIP, n = 7). Bone marrow derived stromal cells (BMSC) from adult male rats were transfected with HGF, instilled intratracheally into bleomycin injured rat lungs and analyzed 7 and 14 days later.

**Results:**

In UIP, HGF was expressed in specific cells mainly located in fibrotic areas close to the hyperplastic alveolar epithelium. HGF-positive cells showed strong co-staining for the mesenchymal stem cell markers CD44, CD29, CD105 and CD90, indicating stem cell origin. HGF-positive cells also co-stained for CXCR4 (HGF+/CXCR4+) indicating that they originate from the bone marrow. The stem cell characteristics were confirmed in HGF secreting cells isolated from UIP lung biopsies. In vivo experiments showed that HGF-expressing BMSC attenuated bleomycin induced pulmonary fibrosis in the rat, indicating a beneficial role of bone marrow derived, HGF secreting stem cells in lung fibrosis.

**Conclusions:**

HGF-positive stem cells are present in human fibrotic lung tissue (UIP) and originate from the bone marrow. Since HGF-transfected BMSC reduce bleomycin induced lung fibrosis in the bleomycin lung injury and fibrosis model, we assume that HGF-expressing, bone-marrow derived stem cells in UIP have antifibrotic properties.

## Introduction

Idiopathic pulmonary fibrosis (IPF) is a progressive disease with a complex pathophysiology and very limited therapeutic options. It is characterized by severe architectural destruction of the lung tissue leading to fibrotic changes and resulting in impairment of gas exchange and finally death due to respiratory failure [1], [2]. The mean survival of patients suffering from IPF is 2–4 years. The recent research to understand the pathogenesis and progression of this lethal disease is focused on a dysregulated wound healing response after repeated injury of the alveolar epithelium [3], [4]. Some evidence suggests that injury of the alveolar epithelium may be a consequence of mechanical stretch caused by alveolar collapsibility [5], [6], [7], [8], [9].

The pathological hallmark of IPF is the pattern of usual interstitial pneumonia (UIP), characterized by heterogeneously distributed fibrotic areas and the presence of fibroblastic foci. Several mechanisms are discussed to contribute to the accumulation of (myo)fibroblasts and the development of fibrosis in the lung. Some recent evidence suggest the transformation of epithelial cells into (myo)fibroblasts by a epithelial-mesenchymal transition (EMT) [10]. There are also reports indicating that fibrocytes which are of mesenchymal origin are a potential source of fibroblasts in the fibrotic lungs [11], [12]. Moreover, recent data indicate the presence of a stem cell pool in the lungs [13]. However, the exact origin and functional role of mesenchymal or resident stem cells with multipotent, self-renewing properties are not known in detail [14], [15].

Several pro- and antifibrotic mediators contribute to the alveolar repair process in UIP lungs. Among them, hepatocyte growth factor (HGF) was shown to have anti-fibrotic properties in an *in vivo* animal model [16]. HGF induces lung epithelial cell proliferation, migration and wound repair and exerts anti-apoptotic properties in lung epithelial cells, but not in fibroblasts [17]. HGF was detected in bronchoalveolar lavage (BAL) and serum of IPF patients [18], however the exact source of HGF in the fibrotic lung is not known. Recent publications showed defective HGF production and secretion by fibroblasts obtained from patients with IPF, indicating that low levels of HGF in the fibrotic lung may contribute to the development of lung fibrosis. Similar findings were also reported in bleomycin injured mouse lungs [19], [20], [21].

We therefore studied the expression and cellular sources of HGF in fibrotic lungs of patients suffering from (UIP) and show that HGF is expressed in specific cells that co-express stem cells markers. HGF-expressing stem cells stain for CXCR4 and therefore originate from the bone marrow, exert multipotent, self-renewing properties *in vitro* and may have a therapeutic potential when administered into the fibrotic lung as we demonstrate in the bleomycin-induced lung injury and fibrosis model in the rat.

## Materials and Methods

### Paraffin embedded lung tissue

Lung tissue sections from patients diagnosed with UIP/IPF (n = 7) according to the ATS/ERS published clinical guidelines [22], [23] were studied. Formalin-fixed and paraffin-embedded lung wedge resections of patients were obtained from the Institute of Pathology, University of Bern. Routine hematoxylin and eosin (H&E), alcianblue as well as elastin-van gieson staining were performed in all patient samples.

### Primary Cell Culture from Human Lung Tissue

Isolation of primary human stem cells was successfully established using lung tissue obtained from surgical lung biopsies performed at the Division of Thoracic Surgery, University Hospital Basel, Switzerland. Primary human stem cells were isolated from lung tissue biopsies as follows: Peripheral lung tissue was cut into small pieces of 1 mm^3^ which were placed into pre-wetted 25 cm2 cell culture flasks. Culture medium, consisting of RPMI supplemented with 10% fetal bovine serum (FBS), 20 U/L penicillin, 20 μg/ml streptomycin and 2.5 μg/ml amphotericin B, was replaced every fourth day. Cells were grown under standard conditions (37°C, 21% O2, 5% CO2). For immunofluorescence staining confluent, non-passaged cells were fixed with 4% formalin (10 minutes), and permeabilized with methanol/acetic acid (3∶1, ice-cold, 10 minutes).

Human BMSC were obtained from the patients undergoing orthopedic surgery at the University Hospital Bern, Switzerland. The cells were grown in Iscove's Modified Dulbecco's Media (IMDM) supplemented with 10%FBS, 1% penicillin/streptomycine (P/S). All experiments were carried out at passage 2–3.

### Ethical approval for patient material

[TIGTER]For collection of paraffin embedded lung tissue and human BMSC, a written informed consent was obtained by the University Hospital, Bern, following the guidelines and approval by the Human Ethics Committee of the University of Bern, Switzerland. Isolation of primary human stem cells using lung tissue obtained from surgical lung biopsies was approved by the Human Ethics Committee of the University of Basel, Switzerland. Written informed consent was obtained from all patients involved in the study.

### Immunohistochemistry

For immunohistochemistry, formalin-fixed human lung tissue sections were deparaffinized in a xylene series and rehydrated through a decreasing ethanol series. The slides were pre-treated by microwave in citrate buffer (100 mM, pH 7.0) for 10 minutes, washed three times with 1xTBS+0.1% tween and incubated overnight at 4°C with one of the following antibodies at the appropriate concentrations against: HGF (1∶100) (R&D Systems, Abingdon, UK), SP-C (1∶100) (Santa Cruz CA, USA), c-Met receptor (1∶50) (Calbiochem USA) or Vimentin (1∶50) (Sigma Aldrich USA), CXCR4 (1∶500) (Abcam USA), a human mesenchymal cell marker panel (CD44 (1∶1000), CD45 (1∶2000), CD90 (1∶500), CD29 (1∶500) and CD105 (1∶2000) (Abcam USA)), αSMA (1∶100) (Sigma Aldrich, USA), myeloperoxidase (1∶500) (Dako USA), HMB45 (1∶50) (Abcam USA) and stained with EnVisionTM+ System-HRP (DAB) (Dako USA). For co-staining, slides were incubated with different combinations of antibodies∶ HGF and SP-C, HGF and αSMA, HGF and CD68, HGF and DC sign, HGF and CD44 and HGF and myeloperoxidase,CD29 and CXCR4; and stained with EnVisionTM DuoFLEX Doublestain (Dako USA), where DAB was used for HGF and permanent red for other antibodies (Dako USA). Images were taken using a Leica DM 3000 microscope.

### Immunofluorescence

For immunofluorescence, the slides were pre-treated by microwave in citrate buffer (100 mM, pH 7.0) for 10 minutes and then washed with 1× PBS before incubation overnight at 4°C with primary antibodies as described above. After 1× PBS wash the slides were treated with the respective secondary antibodies at a dilution of 1∶1000 and incubated in moist chamber for 2 hours at room temperature. For double staining the procedure was extended by incubating the slides with the second primary antibody overnight at 4°C in a moist chamber followed by an incubation with the corresponding secondary antibody for 2 hour at room temperature the following day. Double staining was performed using HGF and Vimentin, CD105, Nanog, Oct3/4, CD44,CD 90 and CXCR4 respectively. For triple staining, the slides were incubated with the third primary antibody on the second day at 4°C overnight in a moist box and the sections were then treated with the third secondary antibody for 2 hours at room temperature. The different fluorescent labelled antibodies used were as CY5 (anti-goat), CY3 (anti-rabbit), FITC (anti-mouse or anti-rabbit as needed) (Abcam, USA). The various combinations for triple staining were HGF+SP-C+CD105, HGF+CD105+CXCR4, HGF+CXCR4+Nanog and HGF+CXCR4+ Oct3/4.

### *In vitro* alveolar epithelial wound repair assay

Human A549 alveolar epithelial-like cells [American Type Culture Collection (ATCC), Rockville, MD] and BMSC were cultured in IMDM supplemented with 10% FBS and 1% P/S. A549 with Dil (red) and BMSC with DiO (green) were labelled with Vybrant Multicolor cell labelling kit (Molecular probes, USA). The cells were labelled with appropriate dye at 37°C for 10 min and then washed twice with 1×PBS. After labelling 1×10^5^ BMSC were plated on the opposite side of a 4µm pore size high pore density PET membrane (transwell cell culture insert by Becton&Dickinson, USA) After 6 hours the transwell inserts were put in a 24 well plate and 1×10^5^ A549 labelled cells were plated inside the transwell insert; when the A549 cells achieved confluence, a mechanical wound was created with a pipette tip, and the co-culture system was placed in serum free media. Images of the wound surface were captured at time 0 and after 24 hours using an inverted microscope (Leitz Diavert, Wetzlar, Germany) connected to a digital camera (Nikon Coolpix). Analysis of the wound surfaces was performed using Image J software (NIH, USA) and epithelial wound repair was expressed as percentage of epithelial wound closure after 24 hours. The co-culture were washed in PBS and fixed for 15 minutes at room temperature in 3% paraformaldehyde in PBS. Fixed cells were treated with 0.1 M glycine in PBS and imaged. In *in vitro* experiments rat BMSC were used and the similar experiments were repeated using human BMSC.

### Laser Scanning Microscopy and Image Restoration

A Zeiss LSM exciter with an inverted Zeiss microscope (Axiovert 200M, Lasers: HeNe 633 nm, HeNe 543 nm, and Ar 488 nm) with either a ×40 or a ×63 objective lens (oil immersion, NA  = 1.3) was used. Image processing and visualization was performed using IMARIS (Bitplane AG, Zurich, Switzerland), a three-dimensional multi-channel image processing software for confocal microscopic images. For the visualization of three-dimensional data sets, particularly for the localization of the cells, with relation to other cells the surpass module from IMARIS was used, which provides extended functions: the volume rendering, which displays the volume of the entire data set, or the IsoSurface visualization, which is a computer-generated representation of a specific grey value range in the data set. It creates an artificial solid object to visualize the range of interest of a volume object.

### *In vivo* animal model of bleomycin-induced lung injury and fibrosis

#### Animals

Male Fisher F344 rats (240–280 g) were obtained from Charles River Laboratories GmbH, Sulzfeld Germany. Experiments were performed in accordance to the standards of the European Convention of Animal Care. The study protocol was approved by the University of Bern Animal Study Committee (nr 16/08), using appropriate anesthesia.

#### Instillation of bleomycin

At day one of the protocol, F344 rats (220–240 g) were anesthetized by inhalation of 4% isoflurane in a glass chamber, intubated with a 14 GA i.v catheter (Insyte, Madrid, Spain) and instilled intratracheally with bleomycin (1.28 U/rat) to both lungs. The dosage of bleomycin was based on preliminary experiments showing induction of pulmonary fibrosis with lowest mortality.

### Isolation and culture of rat bone marrow derived mesenchymal stem cells

Bone marrow derived mesenchymal stem cells (BMSC) were isolated from male Fisher F344 rats. The animals were anesthetized by inhalation of 4% isoflurane in a glass chamber, followed by intraperitoneal administration of Thiopental (50 mg/kg body weight) to sacrifice the animals. The femur and tibia from the hind limbs were dissected and skin and muscles were removed. The isolated femur and tibia were placed in 70% isopropanol for 30 seconds and then transferred in fresh 1×PBS. The bones were cut at the ends and flushed with 1ml 1×PBS using a 22G needle, the follow-through was collected in a sterile tube; each bone was flushed 2–3 times with a volume of 1ml each time. After centrifugation the cells were re suspended in IMDM supplemented with 20% FBS and 1% P/S media and plated in 75 cm flasks and placed at 37°C at 5% CO2 incubator. After 3 days the medium was changed, subsequently media was changed every day. For the experiments the cells were used at passage 2–4.

### Flow Cytometry

Cell surface immunophenotype was analyzed by staining the BMSC with phycoerythrin- or fluorescein isothiocyanate (FITC)-labeled monoclonal antibodies against CD29, CD90, CD 45, CD31, CD106 (BD Biosciences), CD44 (Serotec), and antihuman HGF antibody. The cells were harvested, resuspended in PBS to obtain a single cell suspension, washed, and incubated for 45 minutes at 4°C in the dark with the staining antibody. For some experiments, 20 µl FcR blocking reagent per 10^7^ cells were added (Miltenyi). Unbound antibody was removed by washing the cells three times in staining buffer (1% BSA and 0.1% NaN3 in PBS) at 4°C. The cells were then resuspended in staining buffer and analyzed with the FACScan (Becton Dickinson).

### Transfection of Rat BMSC

Rat BMSC were transfected with the plasmid pCikhHGF, human HGF driven by the human cytomegalovirus (CMV) early promoter enhancer as described [21] using the Amaxa nucleofection (Lonza, USA) using U-23 settings following manufacturer's protocol, 5×10^5^ cells were transfected in one cuvette. After transfection the cells were kept in warm media and either further characterized or instilled intratracheally in the bleomycin injured animals.

### Labelling and instillation of BMSC

BMSC and HGF-modified BMSC were stained with Vybrant Multicolor cell labelling kit (Molecular probes, USA), following manufacture's instructions; briefly, the cells were labelled with DiO at 37°C for 10 min and then washed twice with 1×PBS. After labelling, the cells were instilled in the rat lung 7 days after bleomycin instillation. Animals were randomly divided into 4 treatment groups (intratracheal instillation of BMSC or HGF-modified BMSC, each n = 16) (3×10^6^cells/animal) sacrificed at two different time points (day 7 and day 14 after BMSC instillation). Six animals from each group at each time points were subjected to stereological analysis, and ten animals were used for histology and collagen assays. Additionally two control groups (intratracheal instillation of media control IMDM, n = 5, or instillation of fibroblasts n = 5 and were sacrificed at two different time points (day 7 and day 14 after control media instillation). The animals were anesthetized as above and intubated with an 14GA i.v catheter (Insyte, Madrid, Spain). 3×10^6^ cells or IMDM control media were instilled intratracheally in a volume of 500 μl.

## Assessment

At day 7 and 14 after BMSC and HGF-modified BMSC instillation, the animals and control animals were anaesthetized as described above. Thiopental (50 mg/kg body weight) was administered intraperitoneally and the animals were ventilated via a tracheostomy using the Harvard Rodent Ventilator with FIO_2_ = 1.0, a frequency of 100 breaths/min, and a tidal volume of 10 ml/kg. Subsequently the pulmonary vessels were flushed with 20 ml of 0.9% saline under pressure of 20 cm H_2_O. The heart-lung block was explanted and tissue samples collected for further analysis.

### Histology and Ashcroft scoring

Routine haematoxylin and eosin staining was performed with formalin-fixed tissue sections. In order to evaluate the extent of pulmonary fibrosis, the scoring system of Ashcroft [24] was used. Briefly, a score ranging from 0 (normal lung) to 8 (total fibrosis) was given for each of five randomly chosen microscopic fields and the mean score of all fields was calculated.

### Hydroxyproline Assay

Lungs were analysed for collagen content as initially described by Woessner [25]. The lungs were excised and snap frozen after having measured the wet weight. The frozen lungs where homogenized and 1 mL of the homogenate was treated with 10% trichloroactic acid (TCA), hydrolyzed with 6 M hydrochloric acid (18 hours at 110°C) and adjusted to pH 7.0. Oxidation was initiated by incubation with 1 ml of chloramin T-reagent for 20 minutes at room temperature and stopped by addition of 1 ml of 3.15 M perchloric acid. After incubation with Ehrlich reagent (p-dimethylaminobenzaldehyde added to methyle cellusolve) for 20 minutes at 55–65°C the absorbance of each sample was measured at 557 nm. A standard curve was generated using known concentrations of reagent grade hydroxyproline (Sigma, USA).

### Stereological assessment

#### Fixation, sampling and processing

For stereological analysis 6 complete lungs per group were taken at 7 day and 14 day after treatment with transfected or non-transfected BMSC resulting in 4 groups. The lungs were instillation fixed with a hydrostatic pressure of 20 cm H2O, using a 4% paraformaldehyde/ 0.1% glutaraldehyde mixture in 0.15 M Hepes buffer. Applying fluid displacement method, the total lung volume (V(lung)) was determined [26]. To give every part of the lung an equal chance of being included in the stereological analysis and thereby representing the whole organ [27] a smooth fractionator sampling method was applied [28]. Seven to nine tissue blocks per lung were prepared for light microscopical stereological analysis. Tissue blocks designated for light microscopy and stereology were subsequently osmicated, immersed in half-saturated watery uranyl acetate, dehydrated in acetone and finally embedded in glycol methacrylate (Technovit® 8100, Heraeus Kulzer, Wehrheim, Germany). From each block the 1st and the 4th section of a consecutive row were cut with a thickness of 1.5 µm, mounted on a slide and stained with toluidine blue or orceine.

#### Stereological analysis

At light microscopic level, stereological analysis was carried out using an Olympus BC2 light microscope equipped with a computer-assisted stereology System (newCAST, Visiopharm, Horsholm, Denmark). All stereological methods used in this study were based on recently published guidelines on quantitative assessment of lung structure [29], [30]. Assessment was based on a cascade sampling design [31]. By means of point counting on the toluidine blue stained sections, the volume fraction of parenchyma within the lung was determined, defined as regions with maintained parenchymal architecture likely to contribute to gas exchange (VV(par/lung)). Among the regions of non-parenchyma, volume fractions of destructed parenchyma, defined as regions where the normal parenchymal architecture could not be recognized any more (VV(des/lung)) were further estimated. Destructed lung tissue included above all fibrotic areas and to a minor amount also inflammatory infiltrations or alveolar edema. The number of ventilated alveoli per lung was determined by means of the physical disector using orceine stained sections [28], [32].

As the total lung volume was known, absolute quantities related to the whole organ could be calculated for each parameter by multiplication with the volume fraction according to a cascade sampling design [30].

### Statistical analysis

Data are presented as mean±SEM. Statistical analysis was performed using non parametric one way ANOVA. Stereological data between different treatment groups after intervention were compared using a student`s t-test. Assessment was performed by means of GraphPad Prism version 4.00 (GraphPad Software, San Diego, CA). The level of significance was p<0.05.

## Results

### HGF-positive cells are present in the fibrotic lung

**Figure 1:**
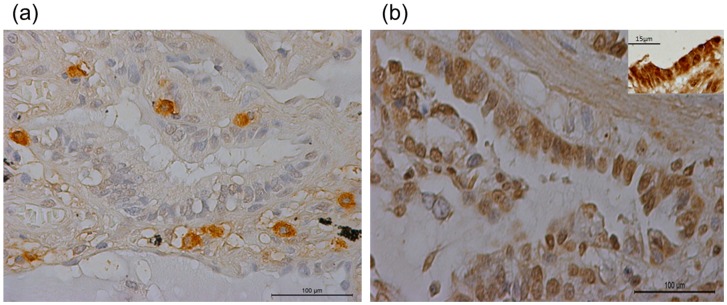
HGF-positive cells are present in the fibrotic lung. Single mononuclear HGF-positive cells were detected in the broadened alveolar septum of UIP, many of them in close proximity of alveolar epithelium (a) Furthermore, alveolar epithelial cells stained positive for c-met, the receptor for HGF (b). magnification (×20) inset higher magnification (×40).

**Figure 2:**
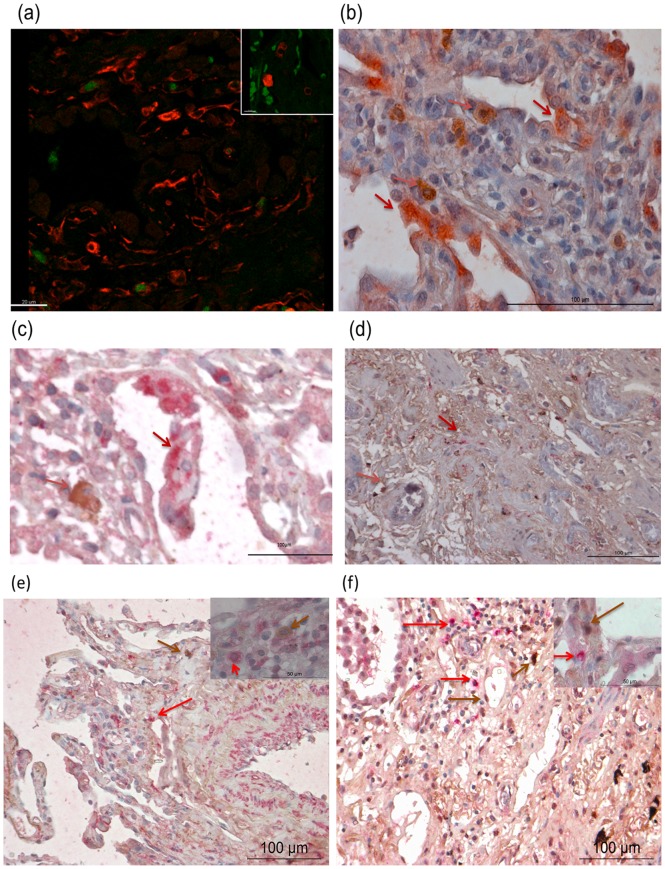
HGF-positive cells do not stain for markers of fibroblasts, dendritic cells, macrophages, myofibroblasts or neutrophils. To further characterize the HGF-positive cells in UIP, the lung tissues were co-stained with markers of the alveolar epithelium, fibroblasts, myofibroblasts, dendritic cells and macrophages using specific antibodies. a) Co staining revealed that the HGF-positive cells (green) did not stain for vimentin (fibroblast marker (red)). (magnification ×20 (inset higher magnification ×40), Figure 2a) b) Co staining with SP-C (pink) shows that the HGF signal (brown) is not present in alveolar epithelial cells (Figure 2b). c) Moreover, no co staining was seen either with CD 68 (macrophage marker) (pink); (Figure 2c) or with DC sign (dendritic cell marker) (pink)(Figure 2d). (magnification ×20). Also the co-stainings with alpha smooth muscle actin to detect myofibroblast (pink) (Figure 2e). (magnification×20 (inset ×100), and anti-myeloperoxidase (pink) to detect neutrophils (Figure 2f). (magnification ×20 (inset ×100), did not show any co staining's. (In Figure 2b-2f HGF is stained brown) (Arrows indicating positive cells).

Distinct individual mononuclear cells with positive cytoplasmic staining for HGF were observed in fibrotic areas of UIP lungs (Figure 1a), many of them in close proximity of the hyperplastic alveolar epithelium that stained positive for c-MET receptor, the receptor for HGF (Figure 1b). Few surfactant protein C (SP-c) positive cells were observed and were hypertropic (Figure S1). Co-staining experiments showed that HGF-positive cells did not stain for vimentin, (Figure 2a, with inset ×100) nor surfactant protein C (SP-C), (Figure 2b), CD-68 (Figure 2c), DC-Sign (Figure 2d), α-SMA (Figure 2e, with inset ×100) or myeloperoxidase (Figure 2f, with inset ×100). These data indicate that HGF-positive cells are neither alveolar type II epithelial cells nor (myo)fibroblasts nor macrophages, dendritic cells or neutrophils. HGF was stained green in immunofluorescence (Figure 2a) and brown in immunohistochemistry (Figure 2b–2f).

### Stem cells expressing mesenchymal stromal markers are present in UIP

**Figure 3:**
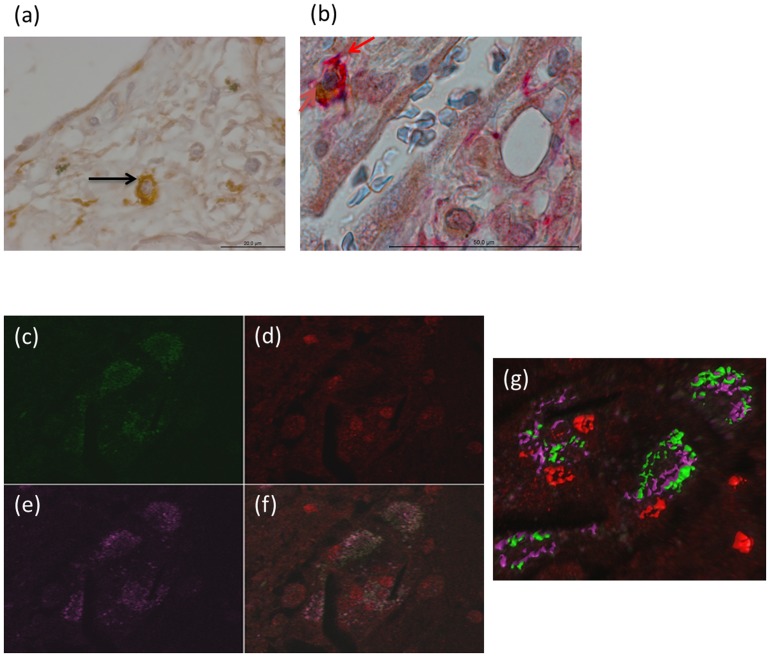
HGF-positive cells stain positive for mesenchymal stromal cell markers. To identify the HGF-positive cells in UIP, the sections were further stained with the mesenchymal stromal cell markers CD44 and CD105. Cells positive for CD 44 (Figure 3a) were seen in varying numbers and homogenously distributed in the tissue section. The cells positive for CD 44 (red) co stained for HGF (brown) (Figure 3b) (magnification ×40). More information with different markers in the supplement. To further localize these cells in relation to alveolar epithelial cells, the triple immunofluroscence staining with HGF (green) (Figure 3c), SPc (red) (Figure 3d) and CD 105 (purple) (Figure 3e) was performed. The cells co expressing CD 105 and HGF, were seen in close proximity to the SP-C positive cells (Figure 3f), (magnification ×40 oil) for better visualization a 3D image rendering was done which clearly demonstrates the co expression of CD105 and HGF and also the close proximity of HGF positive mesenchymal cell to the SPc positive cells (3 g).

Cells positive for the mesenchymal markers CD105, CD29, CD90, CD44 were detected in fibrotic lung tissue obtained from patients with UIP (Figure 3a; CD44), indicating that mesenchymal stem cells are present in UIP. We hypothesized that HGF-positive cells described above may represent a population of mesenchymal stem cells and performed co-stainings for the above described mesenchymal stem cell markers. HGF-positive cells in the fibrotic lung parenchyma stained also positive for CD 105, CD29, CD90, CD44, indicating that HGF-producing cells in the fibrotic lung are mainly from mesenchymal origin (Figure 3b; HGF+CD44). We were not able to detect HGF-positive cells within the area of fibroblast foci or blood vessels, however, triple immunofluorscence (HGF+CD105+SP-C-) showed that HGF-positive stem cells are in close proximity to the alveolar epithelium (Figure 3c-g). These findings suggest that HGF-positive stem cells may specifically interact with the injured and hyperplastic alveolar epithelium in UIP. (Figure S2).

### HGF-positive stem cells in UIP originate from the bone marrow

**Figure 4:**
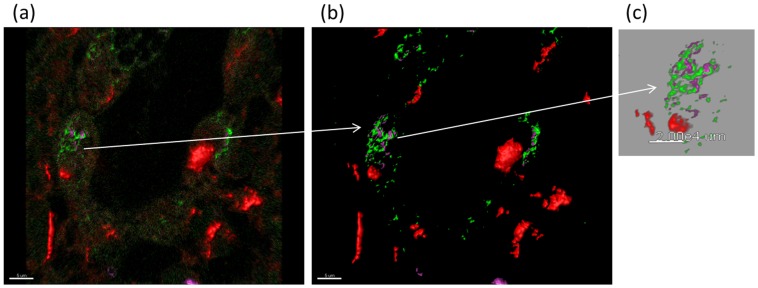
HGF positive cells also stain for CXCR4. Triple immunoflurosence was performed to determine if the CXCR4-positive cells have any correlation with the HGF positive mesenchymal stromal cells, revealed that the cells positive for HGF and CD 105 were also positive for CXCR4. HGF (green),CD 105 (purple) and CXCR4 (red). (Figure 4a), (magnification ×60 oil) the right panel is the 3D construction after image rendering for clarity of the image (Figure 4b), enlarged image for a clear view the colored arrows indicate different staining in the same cell.

Stem cells in the lung may be resident lung stem cells as suggested [13] or mesenchymal stem cells originating from the bone marrow. To determine the origin of HGF-expressing mesenchymal stem cells in UIP, immunostaining for CXCR4, a specific marker for bone marrow derived stem cells, was performed (Figure S3). Triple immunofluorescence staining for HGF, the mesenchymal stem cell marker CD105 and CXCR4 indicates that HGF-expressing stem cells in UIP are positive for CXCR4 and therefore originate from the bone marrow (Figure 4a–c). Interestingly, all HGF-positive cells detected co-stained with CXCR4, indicating that HGF-positive cells originate from the bone marrow (Figure S6).

### HGF expressing mesenchymal stem cells in UIP are pluripotent

To further characterize the HGF-expressing stem cells in UIP, we studied the expression of two different markers of pluripotency, OCT3/4 and NANOG. Triple staining with HGF, CXCR4 and NANOG or Oct 3/4 showed a strong co-staining for all three markers indicating that HGF-expressing stem cells originating from the bone marrow are indeed pluripotent (Figure S4).

### Isolated human mesenchymal stem cells from UIP lungs express HGF and are pluripotent

**Figure 5:**
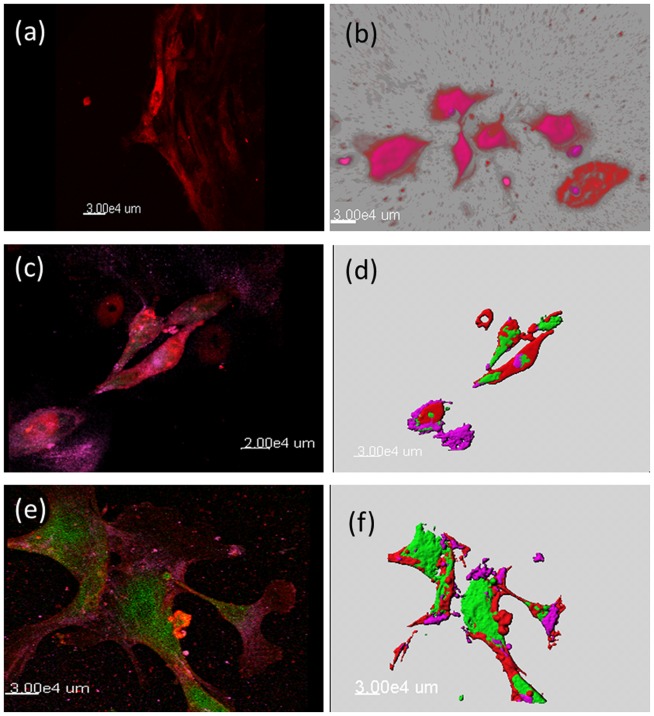
Similar staining pattern was observed in cells isolated from lung biopsies. To further confirm the presence of HGF-secreting pluripotent stem cells of mesenchymal origin, lung biopsies obtained from patients with UIP were grown in culture and the outgrowing cells were stained with markers as in the formalin fixed tissues. The isolated cells stained positive for HGF (red) (Figure 5a), similar as seen in the tissue sections. To further confirm the origin of these isolated cells, co-staining with HGF (red) and CXCR4 (purple) was performed and the similar pattern of costaining was observed (Figure 5b). Furthermore, a triple immunofluroscence staining with HGF (green), CD105 (purple) and CXCR4 (red) was also observed (Figure 5c) in the cells derived from lung biopsies of IPF patients. 3D reconstruction for better visualization (Figure 5d). Moreover, HGF (green) and CXCR4 (red) positive cells also co stained for Oct4 (purple) (Figure 5e), 3D reconstruction for better visualization (Figure 5f) (magnificationx40 oil).

A cell population isolated from surgical lung biopsies from UIP lungs that was negative for epithelial (surfactant protein A) and fibroblast (Vimentin) markers stained positive for HGF (5a) and for the mesenchymal stem cells marker CD105 (Figure 5b). To further characterize the isolated primary cells from UIP lungs with mesenchymal stem properties, we stained the cells for HGF and CXCR4 *in vitro* (Figure 5c, 5d) and show that the isolated cells have similar properties as the described HGF-expressing cells in UIP in vivo, that is are co-staining for HGF and CXCR4 (Figure 5c and 5d). Moreover, the isolated cells also stained positive for OCT3/4 and NANOG (red) (HGF green, CXCR4 purple) (Figure 5e, 5f), confirming the pluripotency of these cells.

### BMSC in the bleomycin lung injury and fibrosis model

To further study the role of HGF-expressing BMSC in the fibrotic lung, we isolated and cultured BMSC from rats and instilled them intratracheally in bleomycin-induced fibrotic lungs after transfection with pCiK-HGF (human hepatocyte growth factor under control of early CMV promoter enhancer).

Before instillation of BMSC *in vivo*, we characterized the BMSC *in vitro* and show a strong staining for CD90, indicating their undifferentiated state. BMSC stained positive for other mesenchymal markers like CD29, CD44, and CD106, whereas labelling for CD45 and CD31 (markers for haematopoetic and endothelial cells) was negative, indicating that BMSC used in passage 2–4 were indeed mesenchymal (Figure S5). After transfection of BMSC with pCikhHGF, FACS analysis revealed that 71.5% of the BMSC stained positive for hHGF, indicating a high transfection rate (Figure S5). After 24 hours in culture, hHGF levels were 17890±4783 ng/ml in conditioned media of transfected cells, indicating that transfected cells secrete biologically relevant amounts of HGF.

### Effects of BMSC and HGF modified BMSC on in vitro alveolar epithelial wound repair

Wounded A549 monolayer growing in a co-culture system with BMSC or HGF-modified BMSC were analysed and compared to wounded A549 monolayer without BMSC as control. Alveolar epithelial repair in vitro was markedly increased in presence of BMSC at 24 hours (wound closure 97.73+/−3.99% vs 24.14+/−0.82% without BMSC, p = 0.0021). In presence of hHGF modified BMSC, alveolar epithelial wound closure was further increased compared to non-transfected BMSC (99.43+/−0.42% (p<0.0001).

### BMSC transfected with hHGF attenuate bleomycin induced lung fibrosis

**Figure 6:**
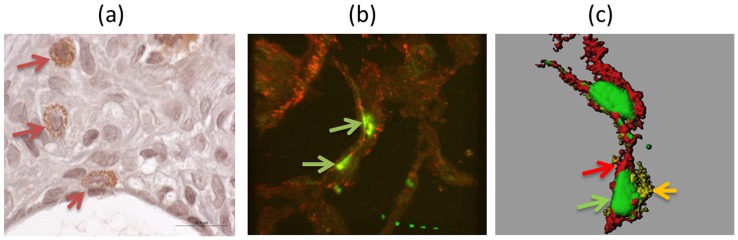
HGF modified BMSC instilled in the bleomycin induced rat lung were seen in the lung parenchyma. The HGF-positive cells were distinctly visible in the rat lung parenchyma by immunohistochemistry (brown) (Figure 6a) (magnification ×60), triple immunofluroscence showing BMSC (DiO labeled, green), SP-C (red), HGF (pale yellow) (6b), 3D image for better visualization (6c). (magnification ×60 oil)

**Figure 7:**
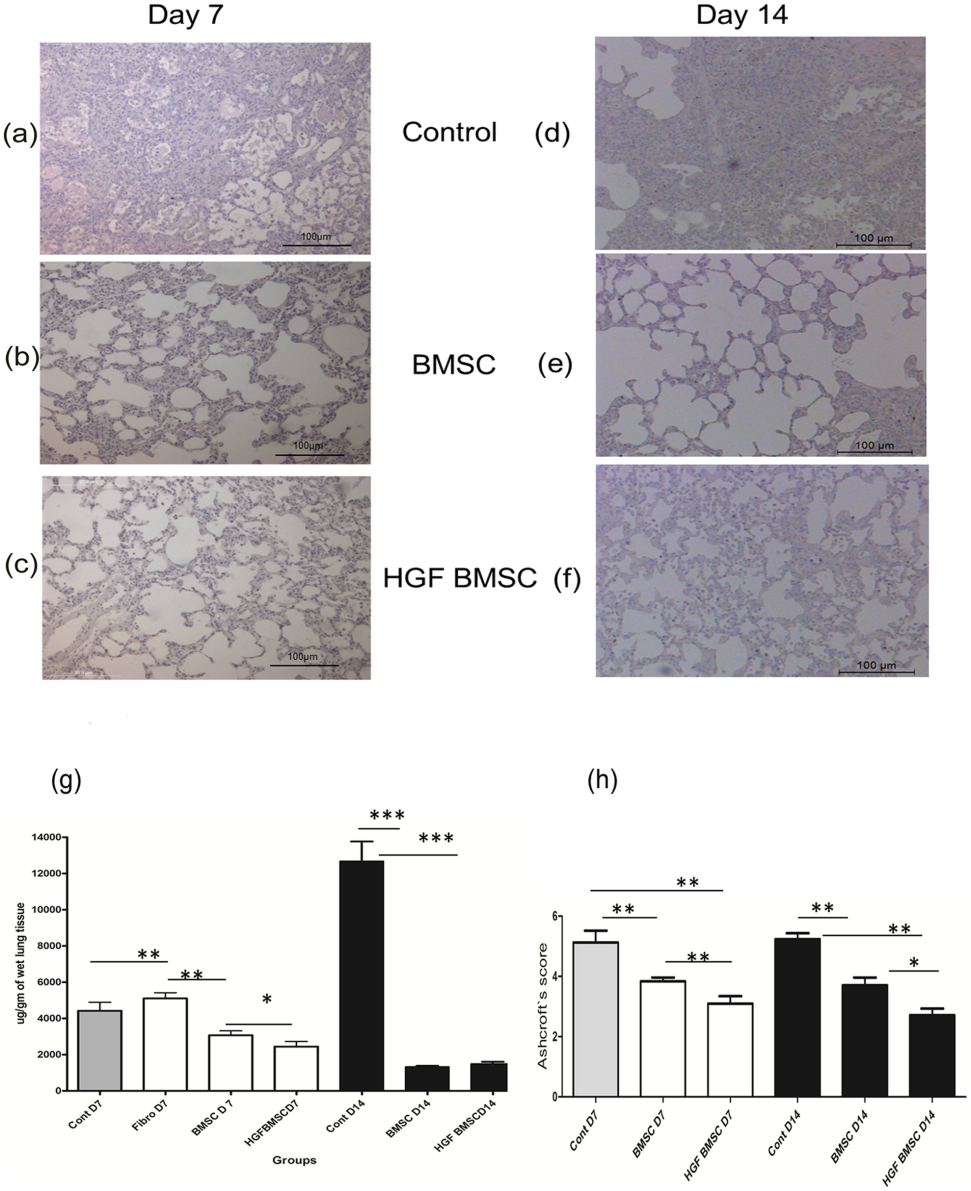
HGF modified BMSC attenuate bleomycin induced fibrosis in rat lung. To further study the possible therapeutic effect of HGF-modified BMSC, the cells were administered intratracheally into bleomycin injured lungs of the adult male Fischer (F344) rats. Seven days after instillation of HGF modified BMSC the lungs were assessed and showed marked improvement in the histological pattern at day 7 (Figure 7c), compared to control animals (Figure 7a) and the group treated with BMSC only (Figure 7b) furthermore when the animals were sacrificed 14 days after HGF modified BMSC instillation there was slight improvement (Figure 7f) compared to control (Figure 7d) and BMSC only group (Figure 7e) in the histological grading which was confirmed with improved Aschroft score (Figure 7h) and reduced collagen content as measured by hydroxyproline assay (Figure 7 g).

Based on our *in vitro* findings on alveolar epithelial wound repair, we studied HGF-modified BMSC in an *in vivo* bleomycin induced lung injury and fibrosis model. Seven days after bleomycin injury, BMSC transfected with pCiKHGF were intracheally instilled (n = 10). Medium alone (n = 5), BMSC without HGF transfection (n = 10) and fibroblast instilled animals (n = 5) served as controls. Seven days after BMSC treatment, distinct HGF positive cells were seen in the rat lung by immunohistochemistry (Figure 6a), furthermore triple immunofluroscence elucidated that the instilled transfected BMSC engrafted themselves in the injured lung and also stained positive for HGF (Figure 6b, 6c). The lung architecture was improved in the treated group as compared to the control group as shown by H&E staining (Figure 7a–f). The hydroxyproline content of the lung treated with BMSC was reduced compared to medium and fibroblast controls (3066±377 µg/mg (BMSC group) vs. 4421±469 µg/mg (medium control), p<0.05 and 5100±315.5 μg/mg, respectively (fibroblast control)). In animals treated with HGF-modified BMSC, the hydroxyproline content was even further reduced to 2446±277 ug/mg (p<0.05 compared to BMSC without HGF modification), indicating that HGF-modified BMSC reveal even more intensive antifibrotic properties compared to unmodified BMSC (Figure 7 g). The Ashcroft score in fibrotic lungs treated with HGF-modified BMSC was lowest compared to unmodified BMSC or medium control (Figure 7h), confirming our results obtained with hydroxyproline measurements showing most reduction of bleomycin-induced fibrosis after treatment with HGF modified BMSC (Ashcroft score 3.09±0.2 after HGF-BMSC, 3.83±0.1 after BMSC, 5.12±0.39 after medium control treatment and 4.614±0.34 in fibroblast treated group, p<0.05). Moreover, when the animals were sacrificed 14 days after treatment the values of hydroxyproline were further reduced in the BMSC group at day 14 (1327±51.18 ug/mg vs 126585±1082 ug/mg in the medium control group), however there was no difference in the collagen content between BMSC alone and HGF modified BMSC (1487±110.1 ug/mg). The Ashcroft score in the HGF-BMSC group, however, showed a significant improvement compared to the BMSC group at day 14 (2.714±0.21 vs 3.709±0.25, p<0.05).

### Stereological data assessed from fibrotic lungs treated with HGF-expressing BMSC

**Figure 8:**
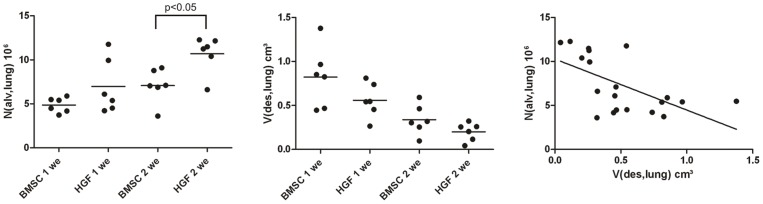
Increased number of open alveoli and reduced volume of destructed lung parenchyma was observed by unbiased stereological analysis. Stereological data of total numbers of alveoli per lung (N(alv,lung)) (Figure 8a) and the total volume of destructed lung parenchyma per lung (V(des,lung)) (Figure 8b). A negative correlation between the number of alveoli and the volume of destructed lung parenchyma can be established (Figure 8c) p<0.01, r = −0.59.

Stereological data (individual data and mean) are illustrated in Figure 8. The number of ventilated (open) alveoli was higher in animals after treatment with HGF-transfected BMSC compared to treatment with non-transfected BMSC. (Figure 8a). The total volume of destructed lung tissue on the other hand was slightly, but not significantly reduced in the HGF transfected BMSC groups compared to BMSC groups at both time points (Figure 8b). Regardless whether BMSC were transfected with HGF or not, a decrease of the total volume of destructed lung tissue was observed. A significant negative correlation between the number of open alveoli and the total volume of destructed lung tissue volume was demonstrated (Figure 8c, r = −0.59, p<0.01). The lower the volume of destructed lung tissue, the higher the number of ventilated alveoli. Taken together, these data are in line with the Ashcroft score indicating that transfection of BMSC with the human HGF-gene enhances the antifibrotic properties and the potential to restore the normal lung architecture.

## Discussion

In this study we show that pluripotent mesenchymal stem cells originating from the bone marrow are present in UIP lungs and express HGF. HGF-modified BMSC reduce pulmonary fibrosis in the bleomycin induced lung injury and fibrosis model, indicating that HGF-transfected BMSC have enhanced regenerative properties and an increased potential to restore normal lung architecture as clearly demonstrated by Ashcroft score and the increased number of open alveoli. We therefore conclude that HGF-positive stem cells detected in UIP may rather be antifibrotic and therefore HGF-modified BMSC might represent a novel therapeutic strategy in patients with UIP/IPF in the future.

HGF is a pleiotropic growth factor having mitogenic activities on alveolar epithelial cells [33], contributes to the morphogenesis of the developing lung epithelium [34], has anti-apoptotic properties on the lung epithelium and induces apoptosis of (myo)fibroblasts [35]. Few studies including our own [16], [36] proved that HGF induces alveolar epithelial wound repair *in vitro* and attenuates bleomycin induced lung injury and fibrosis *in vivo*. In patients with IPF, Marchand-Adam S *et al* showed defective HGF secretion from fibroblasts obtained from UIP lungs [37]. In this respect, HGF may present the basis of a promising novel strategy for the treatment of pulmonary fibrosis.

However, in UIP it is not clear if and by which cells HGF is expressed. *In vitro* studies showed that in specific conditions various cell types like fibroblasts [38], alveolar epithelial cells [18], macrophages [39], dendritic cells [40], neutrophils [41], [42], [43], or myofibroblasts [44] can be a source of HGF. In our study we clearly identified specific HGF-positive cells in the fibrotic lung parenchyma of UIP that co-stained with CD29, CD44, CD90, CD105, and CXCR4, confirming that HGF expressing cells are mesenchymal stem cells that originate from the bone marrow.

Over recent years mesenchymal stromal cells (also referred to as stem cells) have gained a lot of attention due to their possible therapeutic potential, which has been demonstrated in various rodent disease models [45]. Stem cells in the lung may either originate from the bone marrow, being recruited to the lung in case of lung injury, or represent endogenous stem cells. Recent studies reported the presence of multipotent, endogenous or lung-resident stem cells in adult human lungs [46]. In contrast to our findings, Lama VN *et al* [46] suggested that lung resident mesenchymal stem cells are endogenous and do not originate from the bone marrow. This data is based on the study of sex chromosome status in the sex mismatched organ transplant recipient. However, the value of the study was limited by the small number of BAL cells studied which may differ from stem cells within the lung parenchyma. In line with our data, Kajstura J *et al* [13] showed that stem cells are present in the human lung, however they did not discuss the origin of these cells neither compared them to pathological lung conditions such as UIP. In our study we analyzed HGF-expressing stem cells in the fibrotic lung parenchyma of UIP that showed positive staining for CXCR4, clearly indicating that they are bone marrow derived since CXCR4 positivity was shown to represent a reliable marker of bone marrow origin [47]. These findings are in agreement to further reports showing that bone marrow stromal cells actively migrate to the site of injury [48], [49], [50], [51], a process that is mediated in part by the CXCR4 – SDF axis [52]. Since HGF-expressing, CXCR4+ cells in UIP did neither co-stain with vimentin nor with α-SMA, we assume that these cells are not fibrocytes that were recently described to play a major role in the pathogenesis of pulmonary fibrosis [53].

In our study HGF+ cells in UIP also expressed Oct3/4 and NANOG, two transcription factors characteristic of pluripotent stem cells. Interestingly, isolated cells from surgical lung biopsies obtained from patients with UIP/IPF were also positive for mesenchymal stem cell markers, expressed HGF and expressed markers of pluripotency (Oct3/4, NANOG), in a similar pattern as observed in paraffin embedded tissue sections.

In order to characterize the role of HGF-expressing stem cells originating from the bone marrow, we performed *in vitro* and *in vivo* studies using BMSC obtained from humans and rats. We transfected BMSC with human HGF (hHGF) and studied the effect of HGF-modified BMSC in an *in vitro* alveolar epithelial wound repair model using human lung epithelial cells. Finally we instilled HGF-modified rat BMSC intratracheally in bleomycin injured rat lungs. These HGF-expressing BMSC increased alveolar epithelial wound repair *in vitro* compared to unmodified BMSC. Moreover, HGF-expressing BMSC significantly reduced bleomycin induced lung fibrosis as shown by hydroxyproline assay, Ashcroft score and stereological data analysis. The repairing and antifibrotic effect of HGF-expressing BMSC was considerably higher compared to unmodified BMSC (or medium control) at day 7 post treatment. However, there was no difference in the total collagen content 14 days after BMSC or HGF expressing BMSC treatment.

To further assess the effects of HGF-expressing BMSC or unmodified BMSC on lung structure, stereological analysis was performed. Both types of cells were associated with the attenuation of pathological alterations with time as indicated by a decline of the volume of destructed lung tissue and an increase in the number of ventilated alveoli. In addition, differences regarding the efficiency to attenuate pathological alterations could be found. The total volume of destructed lung parenchyma was decreased by trend in HGF-modified BMSC as compared to unmodified BMSC both one and two weeks after instillation of these cells. Moreover, the number of ventilated alveoli was significantly higher in lungs treated with HGF-modified BMSC. In contrast to our data a previous study found 10.5 million alveoli in the normal right lung of Fischer 344 rats of similar age [54] indicating that neither HGF-expressing BMSC nor unmodified BMSC were able to completely restore normal lung architecture two weeks after treatment. Alveolar collapse and collapse induration have been reported to be involved in pathogenesis of fibrosing lung diseases [7], [8], [55]. The negative correlation between the number of ventilated alveoli and the total volume of destructed lung parenchyma suggests that in particular HGF-expressing BMSC lead to a re-opening of previously collapsed alveoli [5], [6]. At the ultrastructural level, collapsed alveoli with opposing denuded basal laminae are a common pathology in the bleomycin-model of the rat lung 7 to 14 days after bleomycin-instillation [56]. Suggesting enhanced repair of the alveolar epithelium in this period occurs and might facilitate the re-opening of collapsed alveoli. Bleomycin injury model does not exactly represent the UIP in terms of the onset and progress of fibrosis; however it is the most commonly used model and partly represents the architectural destruction of the lung, and we show a significant repair reduction of fibrosis in the model using HGF-expressing BMSC.

This potential mechanism is supported by *in vitro* co culture data where regeneration of a wounded A549 epithelial monolayer was efficiently achieved by BMSC after HGF-gene transfection. Thus, the increased number of alveoli in the lungs having received HGF-transfected BMSC is in line with the notion that HGF enhances regeneration of the alveolar epithelial lining *in vivo* [57]. Our previous study did not show any evidence of neo-alveolarisation in healthy lungs overexpressing human HGF specifically by alveolar type II cells [54]. However, the number of alveoli per lung is not a static parameter and in the rat lung late alveolarisation has been discussed, meaning that alveolar number may increase after maturity [58]. Thus, faced with higher levels of HGF, neo-alveolarisation might contribute to an increase of the alveolar number reported here.

In conclusion we report the presence of pluripotent, HGF-expressing stem cells in UIP lungs that originate from the bone marrow. Transfection of rat BMSC with the human HGF gene markedly enhanced their potential to restore normal lung tissue structure after bleomycin lung injury and fibrosis, possibly by reopening the previously collapsed alveoli. Our data indicate that both pluripotent stem cells and HGF may have a beneficial effect in lung fibrosis and may represent the basis of a novel therapeutic approach for patients suffering from UIP/IPF.

## Supporting Information

Figure S1 **The lung sections were stained for surfactant protein C (SP-c), the marker for alveolar epithelial type II cells, not too many positive cells were observed, moreover the observed positive cells where hypertrophic.**
(TIF)

Figure S2 **Cells positive for CD 105 (a), CD 29 (b), CD 90 (c), were seen in varying numbers and were distributed at various locations in the tissue section.** Furthermore, the sections were co stained with. Immunofluroscence with CD 105 and HGF also showed the same results (HGF green (d), CD 105 red (e), merged image (f).
(TIF)

Figure S3 **To identify the origin of HGF secreting, mesenchymal stromal cells, the tissue sections were stained with CXCR4.** Cells positive for CXCR4 were present in the lung parenchyma.
(TIF)

Figure S4 **To ascertain if meschenchymal stromal cells of bone marrow origin expressing HGF have pluripotent properties, sections were stained for markers of pluripotency, a triple staining was performed which exhibited that HGF (green) and CXCR4 (red) positive cells were also immunoreactive to Nanog (Purple) (a).** For clear visualization, 3D image is shown (b).
(TIF)

Figure S5 **The BMSC isolated from the rat where characterized with the known mesenchymal markers (a), significantly higher number of rat BMSC were successfully transfected with human HGF (b).**
(TIF)

Figure S6 **To further confirm that the cells present in the lung are originating from the bone marrow, a costaining with various mesenchymal markers and CXCR4 was performed.** A double immunofluroscence CXCR4 (green) and CD 44 (purple) (a), CXCR4 (green) and CD 90 (purple) (b) CXCR4 (brown) and CD 29 (pink) (c), costaining of CXCR 4 with other mesenchymal markers confirm our findings.
(TIF)
