# Supplementary figures and images for: HGF Expressing Stem Cells in Usual Interstitial Pneumonia Originate from the Bone Marrow and Are Antifibrotic
Source: PLoS One. 2013 Jun 19;8(6):e65453. doi: 10.1371/journal.pone.0065453 (PMC3686785; doi:10.1371/journal.pone.0065453)

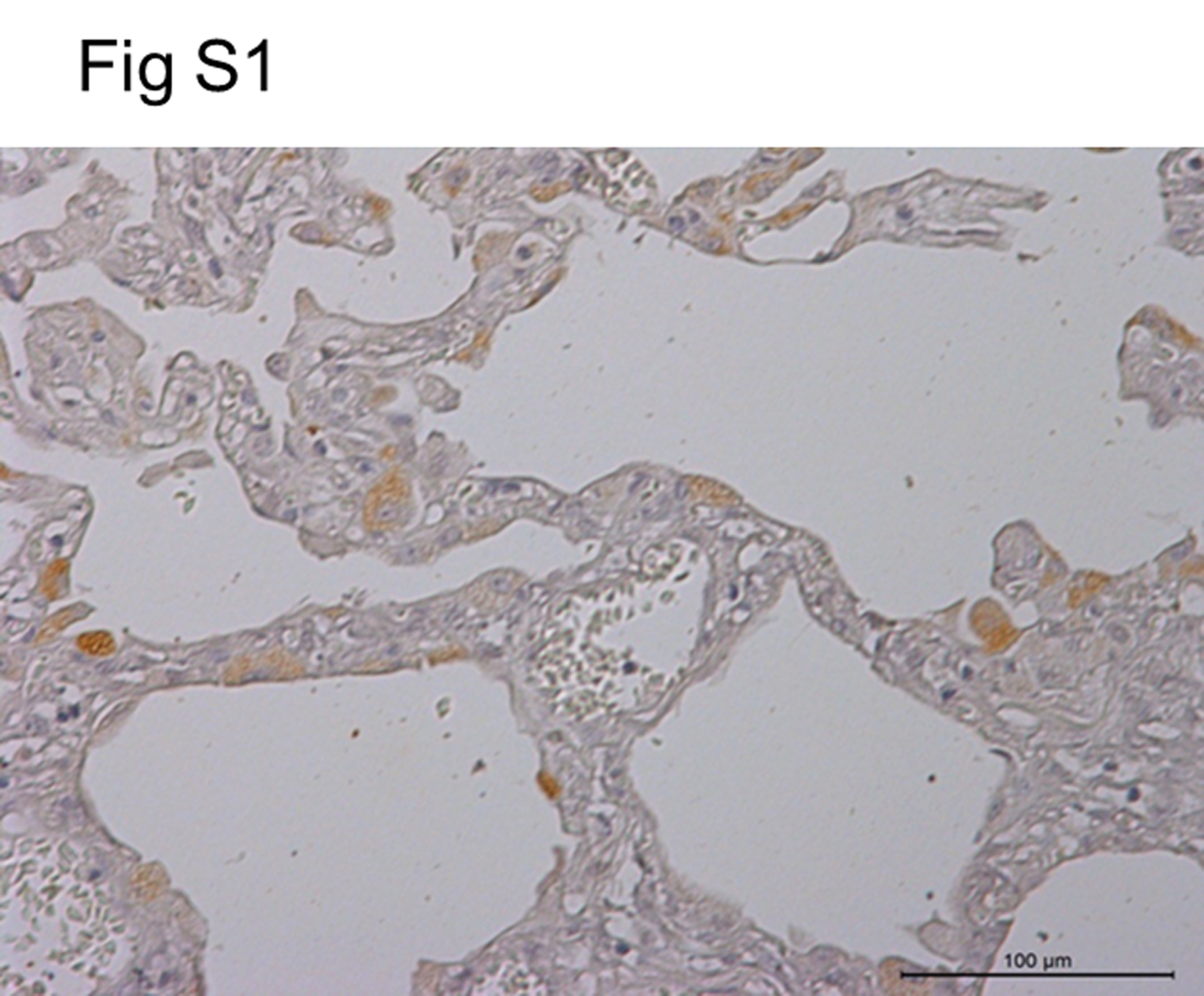

Supplement: Figure S1 — The lung sections were stained for surfactant protein C (SP-c), the marker for alveolar epithelial type II cells, not too many positive cells were observed, moreover the observed positive cells where hypertrophic. (TIF) [file pone.0065453.s001.tif]

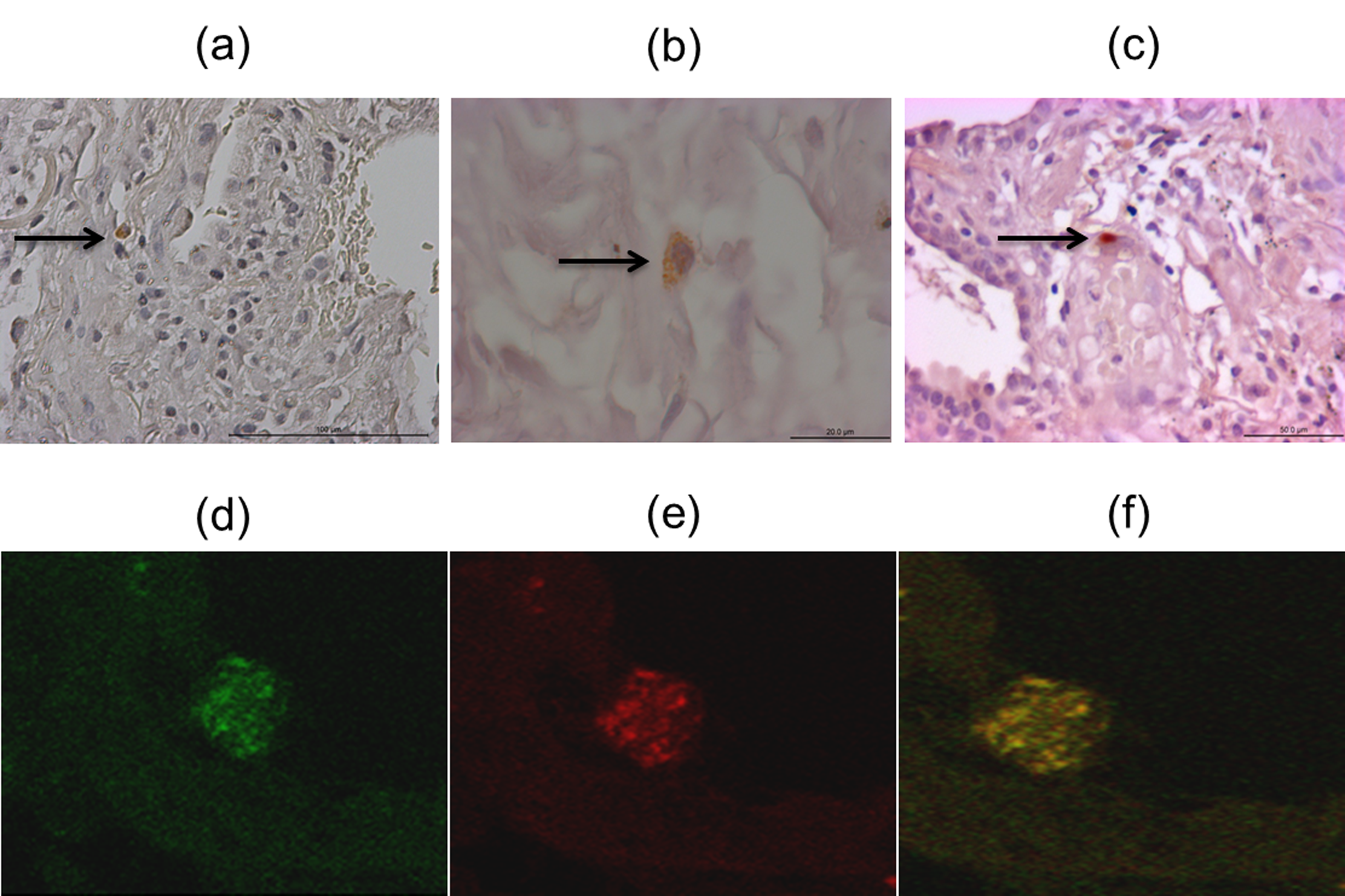

Supplement: Figure S2 — Cells positive for CD 105 (a), CD 29 (b), CD 90 (c), were seen in varying numbers and were distributed at various locations in the tissue section. Furthermore, the sections were co stained with. Immunofluroscence with CD 105 and HGF also showed the same results (HGF green (d), CD 105 red (e), merged image (f). (TIF) [file pone.0065453.s002.tif]

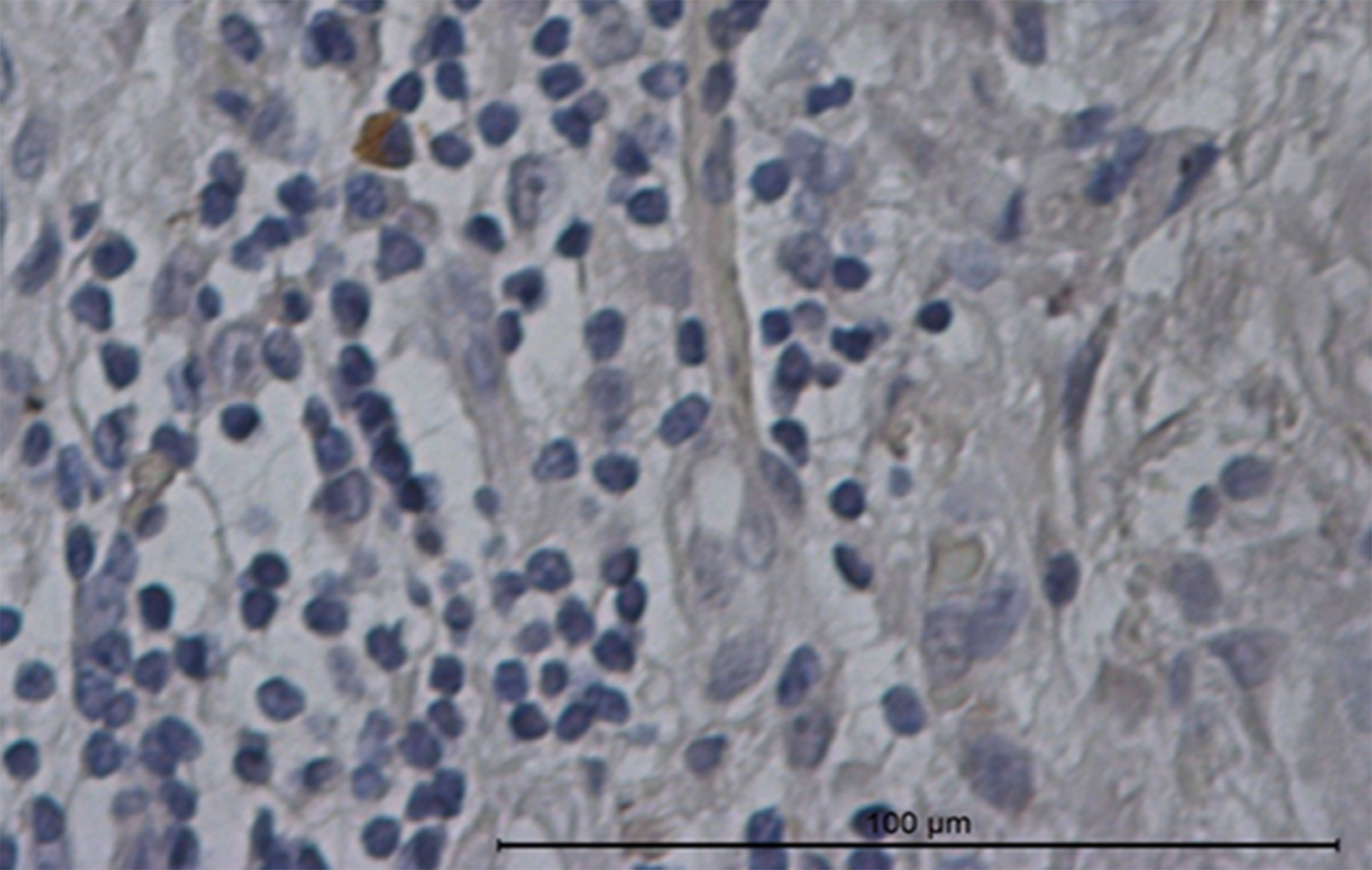

Supplement: Figure S3 — To identify the origin of HGF secreting, mesenchymal stromal cells, the tissue sections were stained with CXCR4. Cells positive for CXCR4 were present in the lung parenchyma. (TIF) [file pone.0065453.s003.tif]

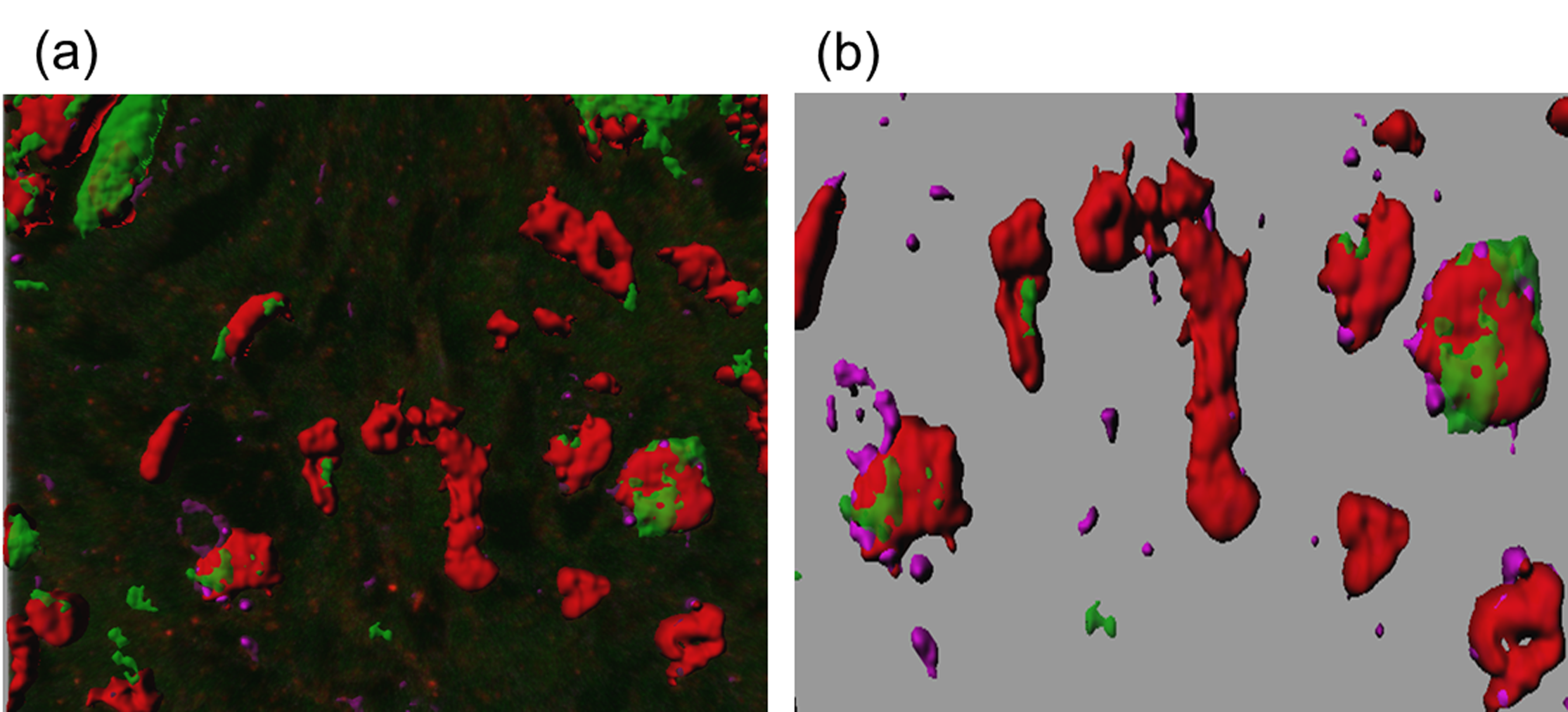

Supplement: Figure S4 — To ascertain if meschenchymal stromal cells of bone marrow origin expressing HGF have pluripotent properties, sections were stained for markers of pluripotency, a triple staining was performed which exhibited that HGF (green) and CXCR4 (red) positive cells were also immunoreactive to Nanog (Purple) (a). For clear visualization, 3D image is shown (b). (TIF) [file pone.0065453.s004.tif]

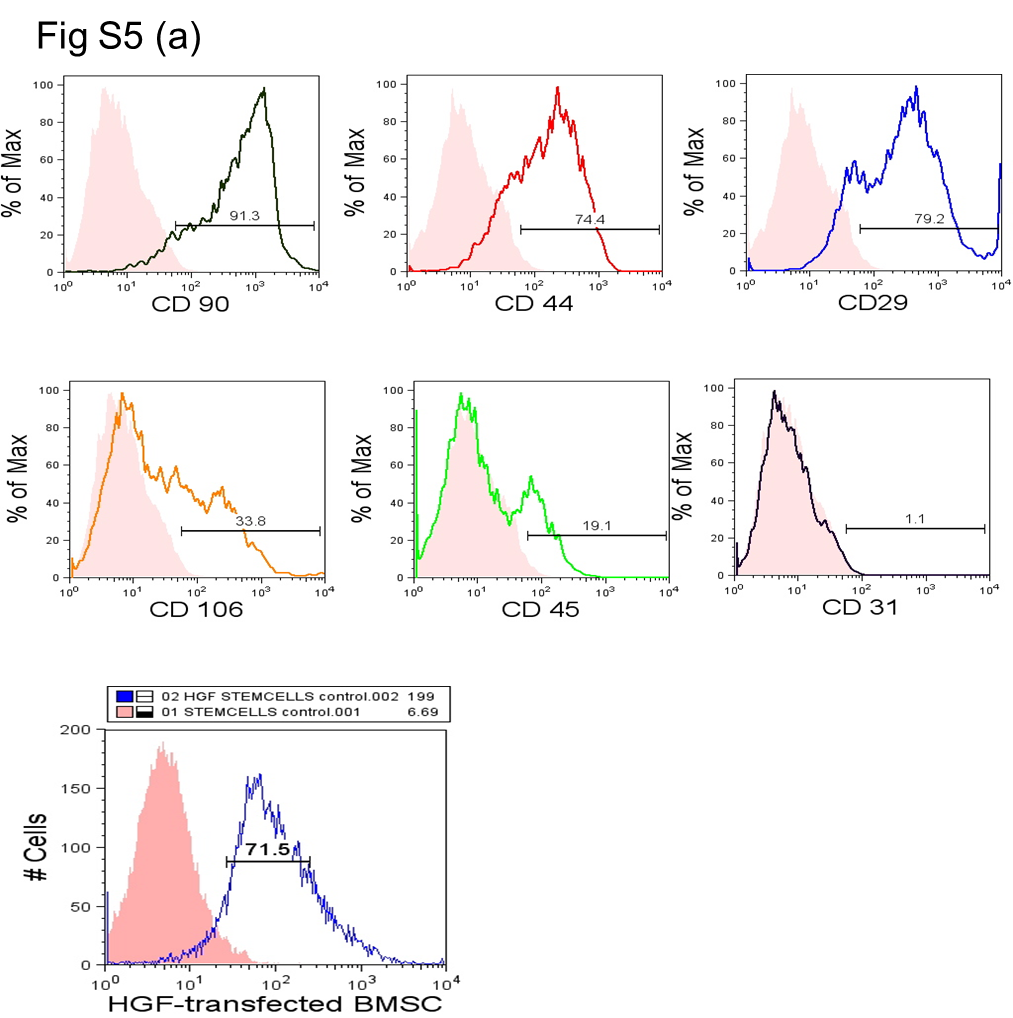

Supplement: Figure S5 — The BMSC isolated from the rat where characterized with the known mesenchymal markers (a), significantly higher number of rat BMSC were successfully transfected with human HGF (b). (TIF) [file pone.0065453.s005.tif]

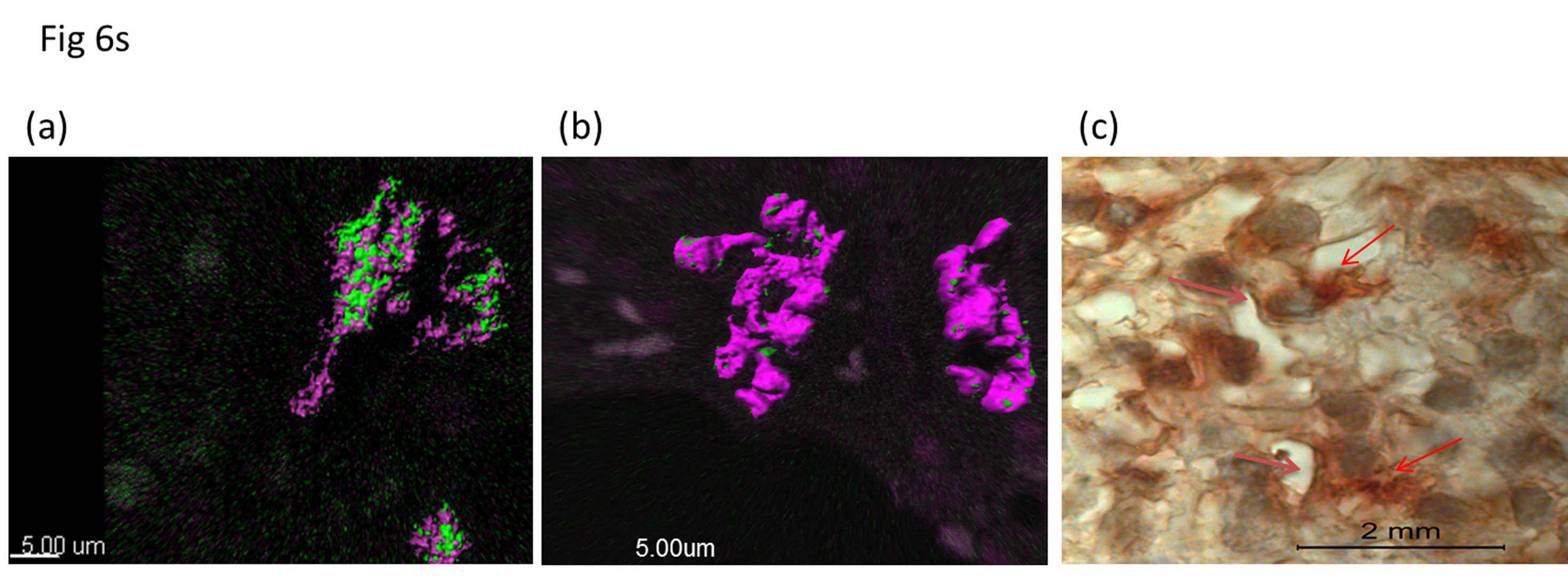

Supplement: Figure S6 — To further confirm that the cells present in the lung are originating from the bone marrow, a costaining with various mesenchymal markers and CXCR4 was performed. A double immunofluroscence CXCR4 (green) and CD 44 (purple) (a), CXCR4 (green) and CD 90 (purple) (b) CXCR4 (brown) and CD 29 (pink) (c), costaining of CXCR 4 with other mesenchymal markers confirm our findings. (TIF) [file pone.0065453.s006.tif]
